# Cyclin D1 as a therapeutic target of renal cell carcinoma- a combined transcriptomics, tissue microarray and molecular docking study from the Kingdom of Saudi Arabia

**DOI:** 10.1186/s12885-016-2775-2

**Published:** 2016-09-30

**Authors:** Sajjad Karim, Jaudah A. Al-Maghrabi, Hasan M. A. Farsi, Ahmad J. Al-Sayyad, Hans-Juergen Schulten, Abdelbaset Buhmeida, Zeenat Mirza, Alaa A. Al-boogmi, Fai T. Ashgan, Manal M. Shabaad, Hend F. NourEldin, Khalid B. M. Al-Ghamdi, Adel Abuzenadah, Adeel G. A. Chaudhary, Mohammed H. Al-Qahtani

**Affiliations:** 1 Center of Excellence in Genomic Medicine Research, Faculty of Applied Medical Sciences, King Abdulaziz University, Jeddah, Saudi Arabia; 2 Department of Pathology, King Abdulaziz University, Jeddah, Saudi Arabia; 3 Department of Pathology, King Faisal Specialist Hospital and Research Center, Jeddah, Saudi Arabia; 4 Department of Urology, Faculty of Medicine, King Abdulaziz University, Jeddah, Saudi Arabia; 5 King Fahd Medical Research Center, King Abdulaziz University, Jeddah, Saudi Arabia; 6 Department of Otorhinolaryngology and Head and Neck Surgery, King Abdulaziz University, Jeddah, Saudi Arabia; 7 KACST Innovation Center for Personalized Medicine, King Abdulaziz University, Jeddah, Saudi Arabia

**Keywords:** Renal cell carcinoma, Cyclin D1, Gene expression profiling, Tissue microarray, Molecular docking, Therapeutic target, Saudi Arabia

## Abstract

**Background:**

Renal cell carcinoma (RCC) is a seventh ranked malignancy with poor prognosis. RCC is lethal at metastatic stage as it does not respond to conventional systemic treatments, and there is an urgent need to find out promising novel biomarkers for effective treatment. The goal of this study was to evaluate the biomarkers that can be potential therapeutic target and predict effective inhibitors to treat the metastatic stage of RCC.

**Methods:**

We conducted transcriptomic profiling to identify differentially expressed genes associated with RCC. Molecular pathway analysis was done to identify the canonical pathways and their role in RCC. Tissue microarrays (TMA) based immunohistochemical stains were used to validate the protein expression of cyclinD1 (CCND1) and were scored semi-quantitatively from 0 to 3+ on the basis of absence or presence of staining intensity in the tumor cell. Statistical analysis determined the association of CCND1 expression with RCC. Molecular docking analyses were performed to check the potential of two natural inhibitors, rutin and curcumin to bind CCND1.

**Results:**

We detected 1490 significantly expressed genes (1034, upregulated and 456, downregulated) in RCC using cutoff fold change 2 and *p* value < 0.05. Hes-related family bHLH transcription factor with YRPW motif 1 (HEY1), neuropilin 2 (NRP2), lymphoid enhancer-binding factor 1 (LEF1), and histone cluster 1 H3h (HIST1H3H) were most upregulated while aldolase B, fructose-bisphosphate (ALDOB), solute carrier family 12 (SLC12A1), calbindin 1 (CALB1) were the most down regulated genes in our dataset. Functional analysis revealed Wnt/β-catenin signaling as the significantly activated canonical pathway (z score = 2.53) involving cyclin D1 (CCND1). CCND1 was overexpressed in transcriptomic studies (FC = 2.26, *p* value = 0.0047) and TMA results also showed the positive expression of CCND1 in 53 % (73/139) of RCC cases. The ligands – rutin and curcumin bounded with CCND1 with good affinity.

**Conclusion:**

CCND1 was one of the important upregulated gene identified in microarray and validated by TMA. Docking study showed that CCND1 may act as a potential therapeutic target and its inhibition could focus on the migratory, invasive, and metastatic potential of RCC. Further in vivo and in vitro molecular studies are needed to investigate the therapeutic target potential of CCND1 for RCC treatment.

**Electronic supplementary material:**

The online version of this article (doi:10.1186/s12885-016-2775-2) contains supplementary material, which is available to authorized users.

## Background

Renal Cell carcinoma (RCC) is a major health problem and accounts for approximately 1.5 percent of all cancer deaths [1, 2]. It accounts for about 3 % of all cancers and 2-3 % per year increase in global incidence [1, 3]. For RCC treatment, surgery is the best option at advance stage, however, one third of patients develop metastases even after surgery [4]. At metastatic stage, prognosis is very poor because RCC patients hardly respond to conventional existing systemic treatments and leads to death [5]. RCC treatment is a big challenge without identification of new drug targets and effective remedies. Although previous studies have reported role of gene alterations, their expression and deregulation of molecular signals to be linked with cancer initiation and progression, there still lack of curative therapy for RCC [6–8]. Therefore, identification of a potential drug target and prediction of suitable ligand is crucial for the patients with RCC.

The cyclin D members (D1, D2 and D3) bind to CDKs and are required for the hematopoietic cells proliferation and survival and perform a rate-limiting antiapoptotic function in vivo [9]. Cyclin D1 (CCND1) overexpression is predominantly correlated with early cancer onset, tumor progression, shorter cancer patient survival and increased metastases [10–12]. Induction of vascular endothelial growth factor (VEGF) production by CCND1 promotes oncogenesis by increasing growth and angiogenesis, while downregulation of death receptor, Fas by CCND1 causes chemotherapeutic and apoptosis resistance [13]. Overexpression of CCND1 has been previously reported in many cancers including lung cancers [14], esophageal squamous cell carcinoma [15], head and neck cancer [16], pancreatic cancer [17], pituitary cancer [18], and breast cancer [19].

CCND1 is a proto-oncogene and a good biomarker for tumor progression, found to be deregulated in several cancers, including RCC. CCND1 along with associated cyclins activates cyclin-dependent kinases (CDKs) - CDK4 and CDK6. G_1_-S phase transition during cell cycle, requires phosphorylation of retinoblastoma (Rb) by CDK4 and CDK6. Hyperphosphorylation of Rb allows expression of genes involved in DNA replication and cell division [20–23]. The ability of CCND1 to exhibit oncogenic property and to regulate a critical G_1_-S transition checkpoint by activating CDK4/CDK6, makes it a potential therapeutic target of RCC [24–28].

Alternative or synergistic anticancer therapies using natural compounds and their derivatives (polyphenols, flavonoids, alkaloids, saponins, etc.) have been extensively studied [29]. Rutin is a flavonol glycoside found in many plants, including buckwheat; tobacco; asparagus, green tea etc. and contributes to the antibacterial [30], hepatoprotective [31], neuroprotective [32] and antioxidant [33] properties of the plant. It is structurally very similar to quercitrin and has been used therapeutically to decrease capillary fragility, to protect blood capillaries, and as ingredients of multivitamin nutritional supplements and alternative herbal remedies. It can attach to iron ion, thereby averting its binding to H_2_O_2_ and free radical generation. In addition, rutin acts as an angiogenesis inhibitor and can stall the *VEGF* in vitro; also has potential anticancerous and antiproliferative property [34, 35].

Curcumin commonly known as turmeric is a phytopolylphenol pigment isolated from the plant *Curcuma longa*, and possesses a variety of pharmacologic properties like anti-inflammatory, antineoplastic, antiproliferative, anticancer, apoptosis inducer, chempreventive [36, 37]. It can inhibit the reactive-oxygen species formation, cyclooxygenases (COX) and other metabolic enzymes involved in inflammation; and can disrupt cell signal transduction via inhibition of protein kinase C. It can interact with myriad of biomolecules by covalent and non-covalent binding. The H-bonding and hydrophobic interactions, arising from the aromatic and tautomeric structures in addition to the flexible linker group owe for the non-covalent interactions [38]. Curcumin reportedly suppress cyclin D1 expression by promoting proteolysis and down-regulating its expression and causes inhibition of CDK4-mediated phosphorylation of retinoblastoma protein [39]. It has been reported that curcumin-treated cells show decreased expression of CCND1, resulting in low cell growth rate. This curcumin-induced CCND1 mRNA down-regulation is perhaps mediated by induction of BTG2 as well as inhibition of nuclear translocation of NF-kappaB [40].

In this study, expression profiling of RCC (CEGMR data) identified 1490 significantly differentially expressed genes and molecular pathway analysis predicted alteration in many important cancer related pathways. However, the major finding of this study was identification and tissue microarray based validation of CCND1 as important over-expressed gene/proteins of RCC. Overexpression of CCND1 can trigger cancer by activating many pathways, including Wnt/β-catenin signaling pathway and has been shown to exhibit oncogenic property, making it a potential therapeutic target. We, therefore, attempted docking study to show the therapeutic potential of anticancerous natural ligands (rutin and curcumin) against the identified potential drug target (CCND1).

## Methods

### Patients and samples

The study was executed on RCC patients from Saudi Arabia and resected tissue samples were collected from collaborating hospitals of Jeddah during the period 2010–2014. For gene expression analysis, fresh surgically resected tumor and normal tissue were collected and stored in RNALater (Invitrogen/Life Technologies, NY, USA) till RNA extraction. All patients included in the present study were Saudi in origin and diagnosed with clear cell or chromophobe renal cell carcinoma without any prior chemotherapy or radiotherapy exposure.

### Ethical approval

Local ethical committee has approved this study (08-CEGMR-02-ETH). Patients were included in the present study only after their prior consent.

### RNA extraction and array processing

Qiagen RNeasy Mini Kit (Qiagen, Hilden, Germany) was used to extract total RNA from fresh kidney tissue, Nano Drop 1000 spectrophotometer (NanoDrop Technologies, Wilmington, DE, USA) was used for concentration determination and RNA quality was checked with Bioanalyzer (Agilent Technologies, CA, USA). Out of 20 specimen, only 7 tumor and 5 control samples passed the selection criteria of RNA integrity number (RIN) >5 and were judged fit to be used for array expression analysis. We used Human Gene 1.0 ST GeneChip arrays (Affymetrix, Santa Clara, CA, USA) for transcriptomics studies (Life Technology, Grand Island, NY), interrogating 764,885 probes and 36,079 annotated reference sequences (NCBI build 36). We processed 250 ng RNA of 12 samples using the Ambion WT Expression Kit (Life Technologies, Austin, TX), GeneChip Hybridization, Wash and Stain Kit (Affymetrix, Santa Clara, CA) and GeneChip WT Terminal Labeling and Controls Kit (Affymetrix, Santa Clara, CA). The hybridization of 5500 ng of cDNA was done in a hybridization oven at 45 °C under rotation (60 rpm) for 17 h. After complete processing, the arrays were scanned in the GeneChip Scanner 3000 7G and GeneChip Command Console Software (AGCC) were used to generate probe cell intensity data (CEL files).

### Gene expression analysis

We carried transcriptomic profiling of 12 samples, seven RCC and five normal kidney tissues. To gain confidence with our limited number samples, we performed a comparative analysis with independent expression datasets from NCBI’s GEO database (GSE781, *n* = 34; GSE7023, *n* = 47; and GSE6344, *n* = 40) for confirmation. Affymetrix. CEL files were imported and analyzed using Partek Genomics Suite version 6.6 (Partek Inc., MO, USA). Default settings robust multi-chip averaged (RMA) was used to log-transform data set and for normalization. Analysis of Variance (ANOVA) was applied, and differentially expressed genes (DEGs) were identified with cut off fold change > 2 and *p* value <0.05. Principal component analysis (PCA) was performed to assess overall expression pattern among sample groups, similar samples were grouped together.

### Tissue microarray and immunohistochemistry

Tissue microarrays (TMA) were designed and constructed for 139 primary RCC and 34 normal kidney tissue as previously described [41]. Experienced pathologist reviewed hematoxylin and eosin (HE) slides of RCC and normal kidney tissue. 1.5 mm tissue cores from areas of interest were chosen from donor block(s) and transferred to recipient paraffin block of TMA Master 1.14 SP3 (3D Histech Ltd, Budapest, Hungary). HE staining of TMA slides was repeated to assess basic morphology of slide construction.

Immunohistochemical studies were performed on positive-charged leica plus slides (Leica Microsystems, Wetzler, Germany) mounted with 4 μm of TMA paraffin blocks. Deparaffinisation of sections was done using xylene, followed by rehydration in an automated BenchMark XT immunostainer (Ventana® Medical systems Inc., Tucson, AZ, USA) and pretreatment in prediluted cell conditioning 1 (CC1) solution for an hour. Immunostaining of TMA slides was done by incubating anti-CCND1 antibody at 37 °C for 16 min, followed by washing, counterstaining (with Mayer’s hematoxylin) and mounting using Ventana® Ultraview Universal DAB detection kit. For analysis and interpretation both negative (with tris-buffered saline only) and positive (with primary antibody) control slides were used. Sections were evaluated independently by the pathologist without knowing the clinicopathological characteristics of RCC patients. Immunostainings were scored semiquantitatively from 0 to 4 + .

### Functional and pathway analysis

We performed pathway analyses and Gene ontology (GO) studies for differentially regulated genes in RCC to find associated biological networks and molecular processes, using Ingenuity Pathways Analysis (IPA) software (Ingenuity Systems, Redwood City, CA). Significantly expressed genes with Affymetrix ID, expression level and *p*-value were uploaded into IPA software to identify the most significant altered biological functions and networks. Fisher’s exact test was used to calculate the significance of association between trancriptomic data and canonical pathways of RCC.

### Molecular docking studies

**Fig. 1:**
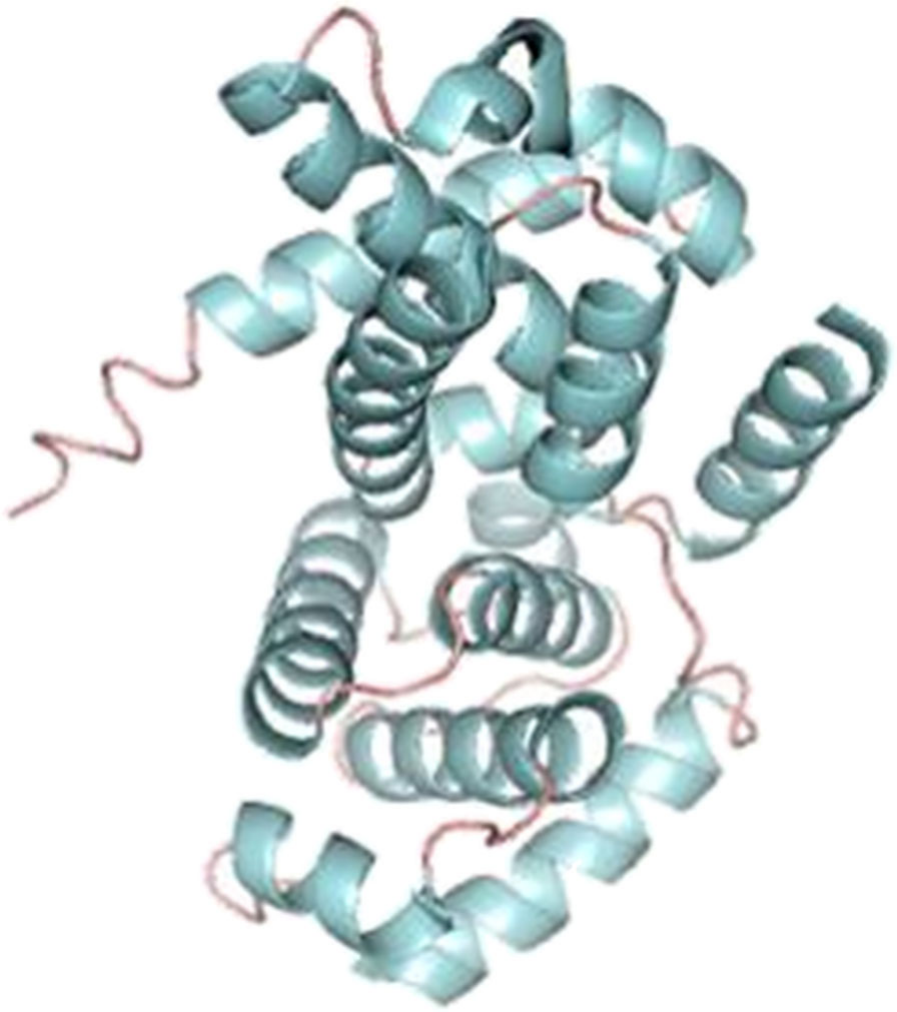
Molecular structure of rutin and curcumin retrieved from NCBI’s PubChem compound database with CID 5280805 and 969516

**Fig. 2:**
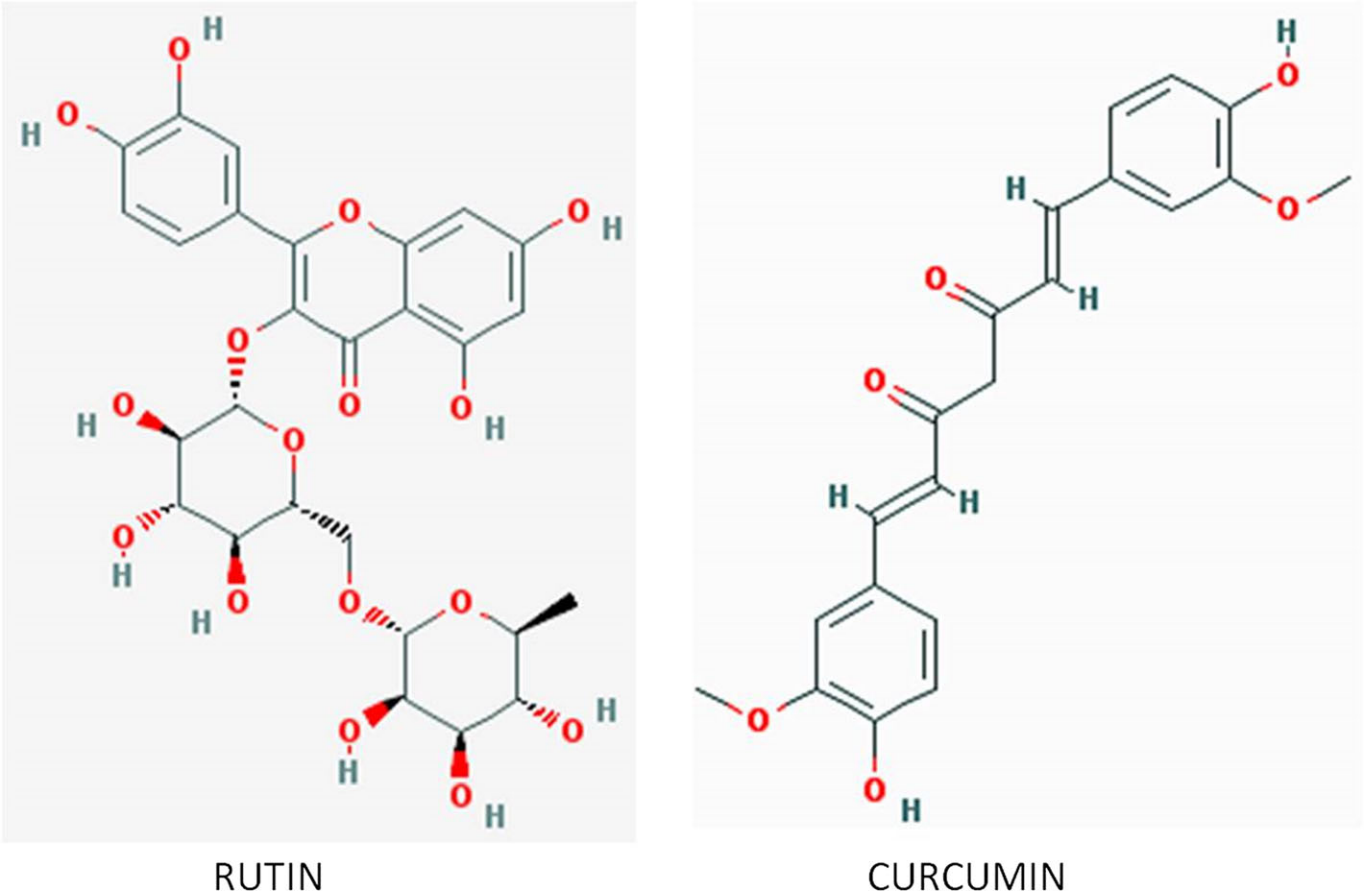
Overall cyclin D1 structure depicted as ribbon diagram (PDB: 2 W96)

The 3-D crystal structure of cyclin D1 was retrieved from RCSB’s Protein Data Bank (PDB) – PDB id: 2w96: Chain A. Structure visualization and illustration was done using PyMol (DeLano Scientific) (Fig. 1). The molecular structure of rutin and curcumin were retrieved from NCBI’s PubChem compound database with CID 5280805 and 969516 respectively (Fig. 2).

Molecular docking was performed using Molecular Docking Server on [42]. The MMFF94 force field geometry optimization method was used for energy minimization of ligand molecule: rutin and curcumin using DockingServer. Gasteiger partial charges were added to the ligand atoms at pH 7.0. Non-polar hydrogen atoms were merged, and rotatable bonds were defined. Rest methodology was followed in sequential manner as previously described [2, 6, 43].

### Supporting data availability

Data series (Accession No. GSE781, GSE7023, GSE6344) used in present study are available at NCBI’s Gene Expression Omnibus database (http://www.ncbi.nlm.nih.gov/geo/).

## Results

This study focused on utilizing transcriptomic profiling to identify biomarkers associated with RCC and conducting molecular docking analysis to assess the interactions between potential target and drugs. We identified CCND1 as important overexpressed gene/proteins of RCC and demonstrated its potential as possible anticancer drug target.

### Identification of differentially expressed genes

**Fig. 3:**
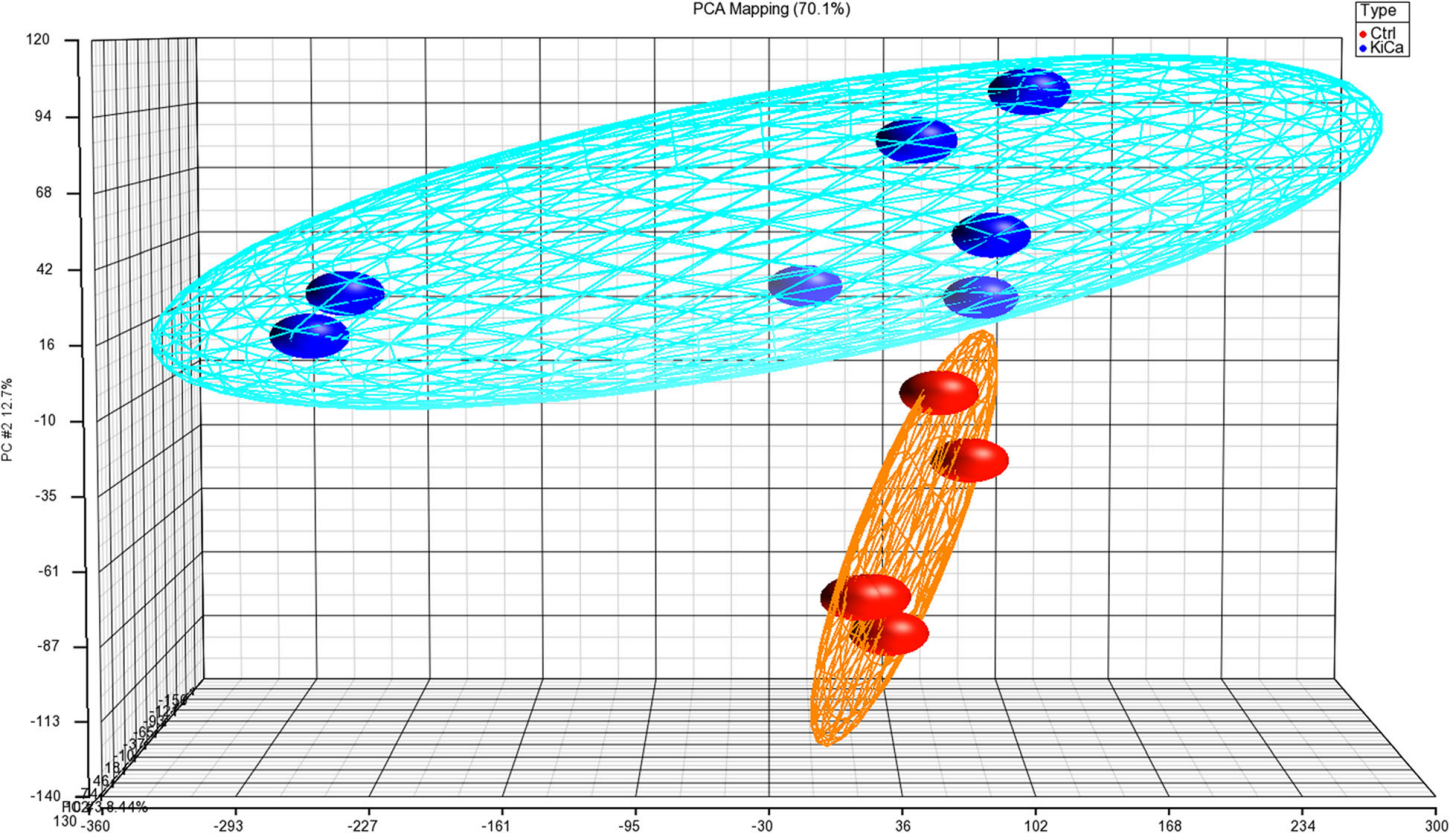
Scatter plot of PCA show grouping of similar type based on genome-wide expression values, as represented as eclipse, where each ball represents one sample. Blue and red is representing RCC and normal kidney tissue

**Fig. 4:**
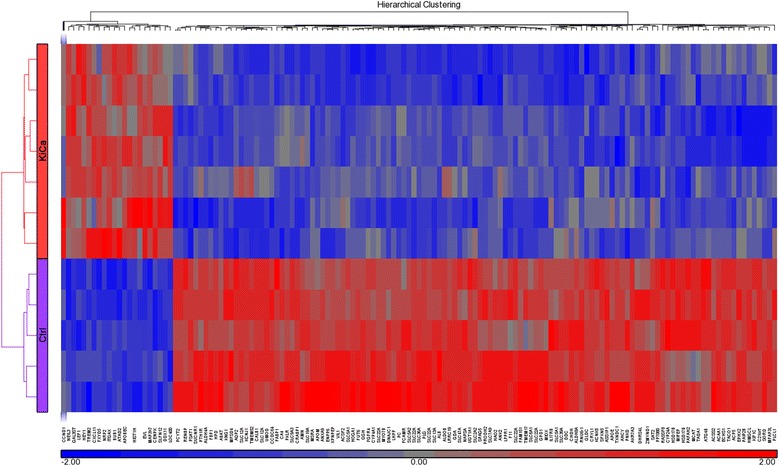
Hierarchical clustering and functional analysis of significantly differentially expressed genes in kidney cancer using Affymetrix Human ST 1.0 array and Partek Genomics suite (ver 6.6)

Three-dimensional scatter plot of PCA demonstrated that RCC and control tissues are distinctly clustered (Fig. 3). We did genome-wide transcription profiling of fresh RCC specimens and identified 1490 differentially expressed genes; 1034 up-regulated and 456 down-regulated using unadjusted *p* value < 0.05 (Additional file 1). Number of differentially expressed genes reduced to 141 (22 up-regulated and 119 down regulated) on applying the stringent condition of false discovery rate with *p* value < 0.05 while keeping all other above parameter same (Fig. 4, Table 1). Hes-related family bHLH transcription factor with YRPW motif 1 (HEY1), neuropilin 2 (NRP2), lymphoid enhancer-binding factor 1 (LEF1), and histone cluster 1 H3h (HIST1H3H) were the most upregulated ones while aldolase B, fructose-bisphosphate (ALDOB), solute carrier family 12 (SLC12A1), calbindin 1 (CALB1) were the most down regulated genes in our dataset. We compared our identified differentially expressed genes list with re-analyzed GEO data series (GSE781, GSE6344 and GSE7023) and identified over-expression of CCND1 in all dataset, thus supporting our result (Table 2).

**Table 1:** Differentially expressed significant genes in RCC

Gene symbol	Gene name	RefSeq	*p*-value	Fold-change
HEY1	hes-related family bHLH transcription factor with YRPW motif 1	NM_001040708	7.88E-06	3.64128
NRP2	neuropilin 2	ENST00000272849	0.00017	3.63215
LEF1	lymphoid enhancer-binding factor 1	NM_001130713	4.04E-05	3.54448
HIST1H3H	histone cluster 1, H3h	NM_003536	3.29E-05	2.87948
ITGAX	integrin, alpha X (complement component 3 receptor 4 subunit)	NM_000887	3.23E-05	2.69367
BUB1	BUB1 mitotic checkpoint serine	NM_001278616	3.35E-05	2.62163
MAP3K7CL	MAP3K7 C-terminal like	NM_001286620	2.81E-05	2.54523
FXYD5	FXYD domain containing ion transport regulator 5	NM_001164605	0.000158	2.45618
HIST1H2AI	histone cluster 1, H2ai	NM_003509	0.000108	2.27918
CENPK	centromere protein K	NM_001267038	6.01E-05	2.27502
CCND1	cyclin D1	**NM_053056**	**0.004789**	**2.25898**
DDX11	DEAD	NM_001257144	0.000178	2.24119
APOBEC3D	apolipoprotein B mRNA editing enzyme, catalytic polypeptide-li	NM_152426	2.61E-06	2.23905
ATAD2	ATPase family, AAA domain containing 2	NM_014109	0.000129	2.18905
HIST1H3F	histone cluster 1, H3f	NM_021018	0.00016	2.14196
TREM2	triggering receptor expressed on myeloid cells 2	NM_018965	0.00021	2.13092
GAL3ST4	galactose-3-O-sulfotransferase 4	NM_024637	1.50E-05	2.11736
LOC400464	uncharacterized LOC400464	AK127420	9.33E-05	2.11281
CXCL11	chemokine (C-X-C motif) ligand 11	NM_005409	0.0002	2.07422
NEIL3	nei endonuclease VIII-like 3 (E. coli)	NM_018248	2.73E-05	2.06874
EVL	Enah	ENST00000553771	5.57E-05	2.06506
SLFN12L	schlafen family member 12-like	ENST00000361112	9.07E-05	2.02832
ALDH4A1	aldehyde dehydrogenase 4 family, member A1	NM_001161504	4.94E-07	−16.1809
SLC22A12	solute carrier family 22 (organic anion	NM_001276326	9.50E-06	−16.4997
SLC47A2	solute carrier family 47 (multidrug and toxin extrusion), me	NM_001099646	5.60E-06	−17.2214
HAO2	hydroxyacid oxidase 2 (long chain)	NM_001005783	0.000192	−17.3322
SLC6A19	solute carrier family 6 (neutral amino acid transporter), me	NM_001003841	0.000195	−18.4585
XPNPEP2	X-prolyl aminopeptidase (aminopeptidase P) 2, membrane-bound	NM_003399	0.000131	−18.9386
CYP4A11	cytochrome P450, family 4, subfamily A, polypeptide 11	XR_246241	7.59E-05	−19.2237
SLC22A6	solute carrier family 22 (organic anion transporter), member 6	NM_004790	5.33E-06	−20.6522
KCNJ1	potassium inwardly-rectifying channel, subfamily J, member 1	NM_000220	8.79E-05	−22.4359
TMEM52B	transmembrane protein 52B	NM_001079815	0.000112	−23.1374
SLC12A3	solute carrier family 12 (sodium	NM_000339	1.14E-06	−27.7638
HPD	4-hydroxyphenylpyruvate dioxygenase	NM_001171993	1.88E-05	−29.7949
SLC5A12	solute carrier family 5 (sodium	XM_006718157	0.000155	−30.9613
KNG1	kininogen 1	NM_000893	4.28E-06	−34.7144
SLC13A3	solute carrier family 13 (sodium-dependent dicarboxylate tra	NM_001011554	1.93E-06	−37.6483
SLC36A2	solute carrier family 36 (proton	NM_181776	4.06E-07	−37.8957
PLG	plasminogen	NM_000301	4.87E-07	−43.0535
SLC22A8	solute carrier family 22 (organic anion transporter), member	NM_001184732	4.50E-06	−45.8474
UMOD	uromodulin	NM_001008389	2.44E-06	−68.9599
CALB1	calbindin 1, 28 kDa	NM_004929	2.29E-06	−78.3947
SLC12A1	solute carrier family 12	ENST00000330289	0.000135	−79.6698
ALDOB	aldolase B, fructose-bisphosphate	NM_000035	2.56E-05	−87.9122

Negative fold change value indicates the downregulation

*bold data shows CCND1 (Cyclin D1) was overexpressed (fold change = 2.258) and statistically significant (*p*-value = 0.00478)

**Table 2:** Expression of CCND1 in Saudi RCC patients (CEGMR dataset) and GEO dataset

Dataset	Sample size	*P*-value	Fold change
CEGMR (own data)	12	0.0047	2.26
GSE781	34	0.0030	2.41
GSE6344	40	1.04 × 10^−9^	4.82
GSE7023	47	6.55 × 10^−5^	3.33

### Validation of CCND1

**Fig. 5:**
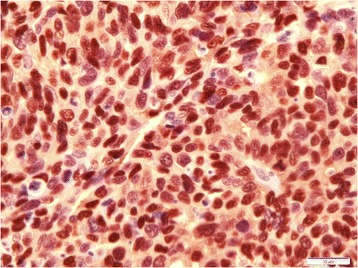
Immunohistochemistry stain for CCND1 show positive staining in RCC (original magnification × 60)

Transcriptomic profiling revealed distinct CCND1 overexpression (FC = 2.26, *p* value = 0.0047). Validation study based on TMA-immunohistochemistry staining showed the positive expression of CCND1 in 53 % (73/139) of RCC cases (Fig. 5).

### Pathways and networks underlying RCC

**Fig. 6:**
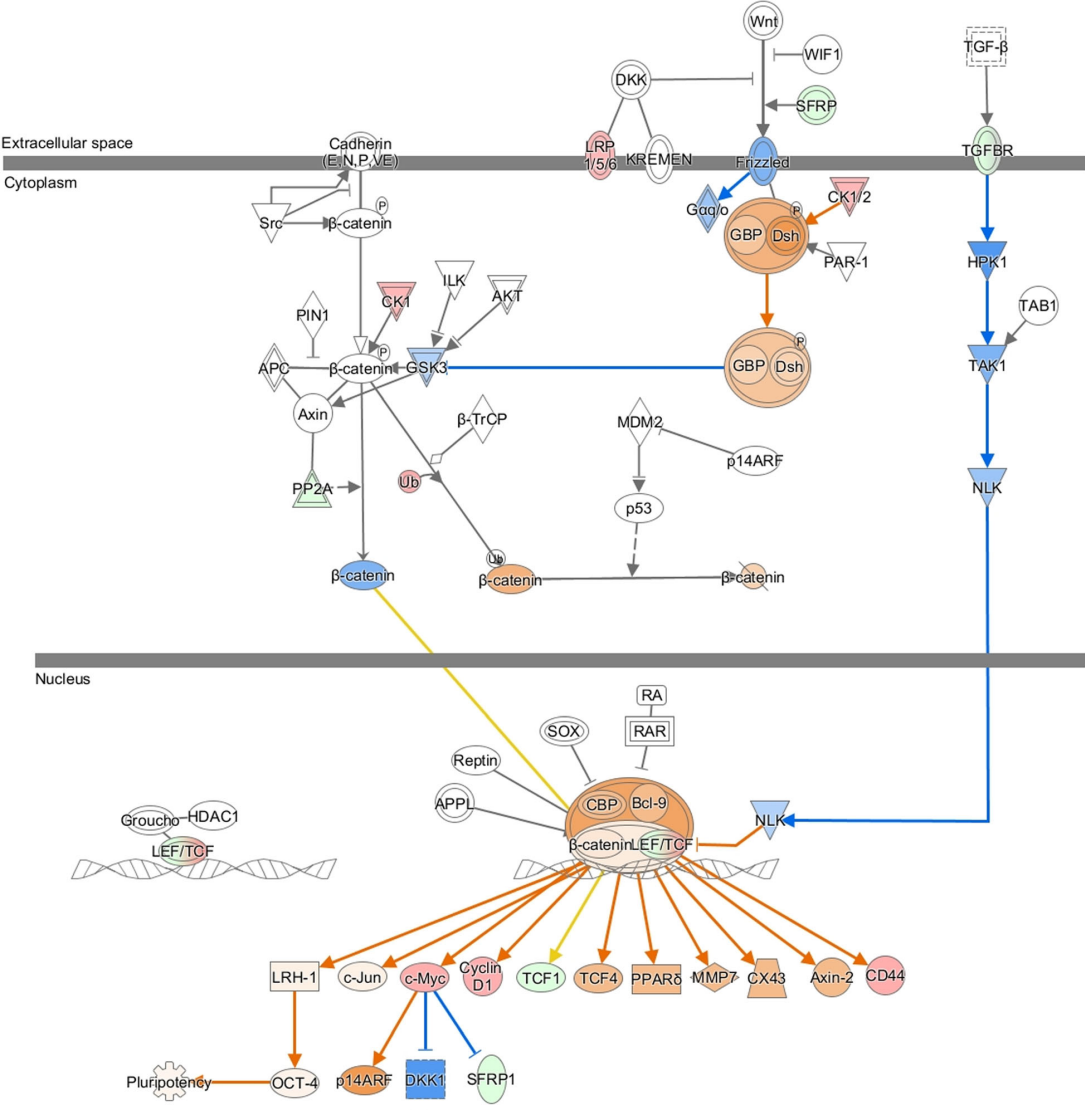
Wnt-β catenin signaling was significantly activated (z-score = 2.53) in RCC based on following differentially expressed genes: cyclin D1 (CCND1, FC = 2.25898), CD44 molecule (CD44, FC = 2.31667), v-myc avian myelocytomatosis viral oncogene homolog (c-Myc, FC = 2.31324), HNF1 homeobox A (TCF1, FC = −2.26661), secreted frizzled-related protein 1 (SFRP1, FC = −4.45838). Red represents overexpression and green underexpression

Pathway analysis of identified DEGs revealed the biofunctions, molecular network and canonical pathways association with RCC (Table 3). Most significantly inhibited pathways were synaptic long term potentiation (z-score = −2.33), NRF2-mediated oxidative stress response (z-score = −2.33), production of nitric oxide and reactive oxygen species in macrophages (z-score = −2.324), and renin-angiotensin signaling (z-score = −2.121). Wnt/β-catenin signaling was significantly activated pathway (z-score = 2.53) involving following genes; cyclin D1 (CCND1, FC = 2.26), CD44 molecule (CD44, FC = 2.31), v-myc avian myelocytomatosis viral oncogene homolog (c-Myc, FC = 2.31), HNF1 homeobox A (TCF1, FC = −2.26), secreted frizzled-related protein 1 (SFRP1, FC = −4.45) (Fig. 6). We found over expression of CCND1 playing important role in regulation of Wnt/β-catenin signaling along with other cancer related pathways like Acute Myeloid Leukemia Signaling, Non-Small Cell Lung Cancer Signaling, PTEN Signaling, Regulation of Cellular Mechanics by Calpain Protease, ErbB2-ErbB3 Signaling, HER-2 Signaling in Breast Cancer, HER-2 Signaling in Breast Cancer, Thyroid Cancer Signaling, Endometrial Cancer Signaling etc. Further extensive molecular pathway analysis may help to better understand the mechanism of RCC initiation, invasion and metastasis.

**Table 3:** Canonical pathways predicted by Ingenuity Pathway Analysis for significant genes differentially expressed in kidney cancer

Ingenuity canonical pathways	-log (*p*-value)	z-score	Molecules
Wnt/β-catenin Signaling	0.271	2.530	CSNK1E,MYC,PPP2R4,TGFBR3,CD44,LEF1,SFRP1,UBC,**CCND1**,HNF1A,ACVR2A,LRP1
Synaptic Long Term Potentiation	0.481	−2.333	PLCB4,PPP1R1A,PPP1R3C,PPP3R1,PRKAR2A,CACNA1C,PLCL1,PLCD4,PRKCZ,PRKCA
NRF2-mediated Oxidative Stress Response	1.5	−2.333	GSTA3,AKR7A2,AKR7A3,GSTM1,GSTM3,NQO2,ABCC2,NQO1,DNAJC19,SOD1,PRKCZ,DNAJC11,AKR1A1,SCARB1,FMO1,GSTA1,AOX1,TXN,PRKCA,EPHX1
Production of Nitric Oxide and Reactive Oxygen Species in Macrophages	0.512	−2.324	PPARA,MAP3K15,APOE,APOM,PPP1R3C,PRKCZ,APOL1,ALB,PPP2R4,CYBA,APOC1,CHUK,APOD,RBP4,PRKCA
Sperm Motility	1.19	−2.309	PLA2G16,SLC16A10,PLCB4,PLA2R1,PRKAR2A,PNPLA3,PLCL1,PLA2G12B,PDE1A,PLCD4,PLA2G7,PRKCZ,PRKCA
Renin-Angiotensin Signaling	0.279	−2.121	ADCY9,GRB2,REN,PRKAR2A,CCL5,PRKCZ,AGT,PRKCA
Nitric Oxide Signaling in the Cardiovascular System	0.27	−1.890	KNG1,CAV1,PRKAR2A,CACNA1C,PDE1A,PRKCZ,PRKCA
Antioxidant Action of Vitamin C	3.01	1.897	PLA2G16,NAPEPLD,PLA2R1,SLC23A3,PLA2G7,GLRX,SLC2A3,PLCB4,SLC23A1,SLC2A2,PNPLA3,CHUK,TXN,PLA2G12B,PLCL1,PLCD4
Aldosterone Signaling in Epithelial Cells	1.34	−1.897	DNAJC12,DNAJC19,PDPK1,HSPD1,HSPA2,PRKCZ,HSPA12A,DNAJC11,PLCB4,SCNN1G,SLC12A1,CRYAA/LOC102724652,SCNN1B,PLCL1,PLCD4,PRKCA,AHCY
Valine Degradation I	10.11	NaN	ECHS1,ABAT,ACADSB,BCKDHB,BCAT1,HIBCH,HIBADH,AUH,DLD,DBT,EHHADH,HADHA,ALDH6A1
Ethanol Degradation II	9.4	NaN	HSD17B10,ADH6,ALDH1B1,ALDH4A1,ACSS1,ALDH9A1,ADH5,ALDH2,AKR1A1,ALDH3A2,ACSS2,ADHFE1,ACSL1,ALDH7A1,DHRS4
Fatty Acid β-oxidation I	9.4	NaN	HSD17B10,ECHS1,SLC27A2,ACAA1,ACAA2,SCP2,ECI2,AUH,ACSL4,IVD,EHHADH,ACADM,HADHA,ACSL1,HADH
FXR/RXR Activation	9.17	NaN	PPARA,KNG1,APOE,PKLR,APOH,ABCC2,SLC22A7,HNF1A,CYP8B1,MTTP,PCK2,SCARB1,SLC10A2,FGFR4,LPL,GC,AGT,APOM,SDC1,UGT2B4,CYP27A1,SERPINF2,APOL1,ALB,FABP6,APOC1,FBP1,G6PC,SLC51B,RBP4,APOD
Serotonin Degradation	8.46	NaN	ADH6,HSD17B10,ALDH4A1,ALDH1B1,UGT3A1,UGT2B4,UGT2B7,UGT1A1,ALDH9A1,ADH5,ALDH2,AKR1A1,SMOX,ALDH3A2,ADHFE1,DHRS4,ALDH7A1,MAOA
Noradrenaline and Adrenaline Degradation	7.86	NaN	ADH6,HSD17B10,ALDH4A1,ALDH1B1,ALDH9A1,ADH5,ALDH2,AKR1A1,SMOX,ALDH3A2,ADHFE1,DHRS4,ALDH7A1,MAOA
Tryptophan Degradation	7.34	NaN	ALDH4A1,ALDH1B1,ALDH2,AKR1A1,SMOX,ALDH3A2,DDC,ALDH9A1,ALDH7A1,MAOA
PXR/RXR Activation	4.04	NaN	PPARA,SCD,GSTM1,ABCB1,ABCC2,PRKAR2A,CES2,HMGCS2,UGT1A1,PCK2,ALDH3A2,GSTA1,G6PC,CYP2B6
Acute Myeloid Leukemia Signaling	0.491	0.816	RUNX1,MYC,GRB2,LEF1,**CCND1**,HNF1A,IDH1
Non-Small Cell Lung Cancer Signaling	0.481	−0.816	GRB2,PDPK1,EGF,ERBB2,**CCND1,**PRKCA
PTEN Signaling	0.662	0.905	FGFR3,GRB2,FGFR4,TGFBR3,PREX2,ITGA5,FGFR2,PDPK1,CHUK,**CCND1,**PRKCZ
Regulation of Cellular Mechanics by Calpain Protease	0.453	−1.000	GRB2,ITGA5,EGF,**CCND**1,ACTN1
ErbB2-ErbB3 Signaling	0.967	−1.890	MYC,GRB2,NRG3,PDPK1,ERBB3,ERBB2,**CCND1**
Aryl Hydrocarbon Receptor Signaling	2.07	NaN	GSTA3,GSTM1,ALDH4A1,ALDH1B1,GSTM3,NQO2,NQO1,ALDH8A1,**CCND1**,ALDH9A1,MYC,ALDH1L1,ALDH1L2,ALDH3A2,GSTA1,ALDH5A1,ALDH6A1,ALDH7A1
HER-2 Signaling in Breast Cancer	1.01	NaN	GRB2,PARD6B,EGF,ERBB3,ITGB8,ERBB2,**CCND1**,PRKCZ,PRKCA
Thyroid Cancer Signaling	0.828	NaN	CXCL10,MYC,LEF1,**CCND1**,HNF1A
Endometrial Cancer Signaling	0.765	NaN	MYC,GRB2,PDPK1,LEF1,ERBB2,**CCND1**
Role of Macrophages, Fibroblasts and Endothelial Cells in Rheumatoid Arthritis	0.615	NaN	CXCL8,FN1,IL1RL1,CXCL12,CCL5,HNF1A,**CCND1**,FCGR1A,PRKCZ,C5,MYC,PLCB4,F2RL1,PPP3R1,LEF1,CHUK,SFRP1,PLCL1,PLCD4,FCGR3A/FCGR3B,TNFSF13B,LRP1,PRKCA,ADAMTS4
Estrogen-mediated S-phase Entry	0.278	NaN	MYC,**CCND1**

*bold data shows presence and importance of CCND1 among identified canonical pathways

### Docking studies

We made a structural attempt to study possible binding of two natural famed ligands with the potential therapeutic drug target, Cyclin D1 for cancer therapeutics. CCND1 protein has a classical double cyclin box domain fold, comprising of 11 alpha-helices [44].

**Fig. 7:**
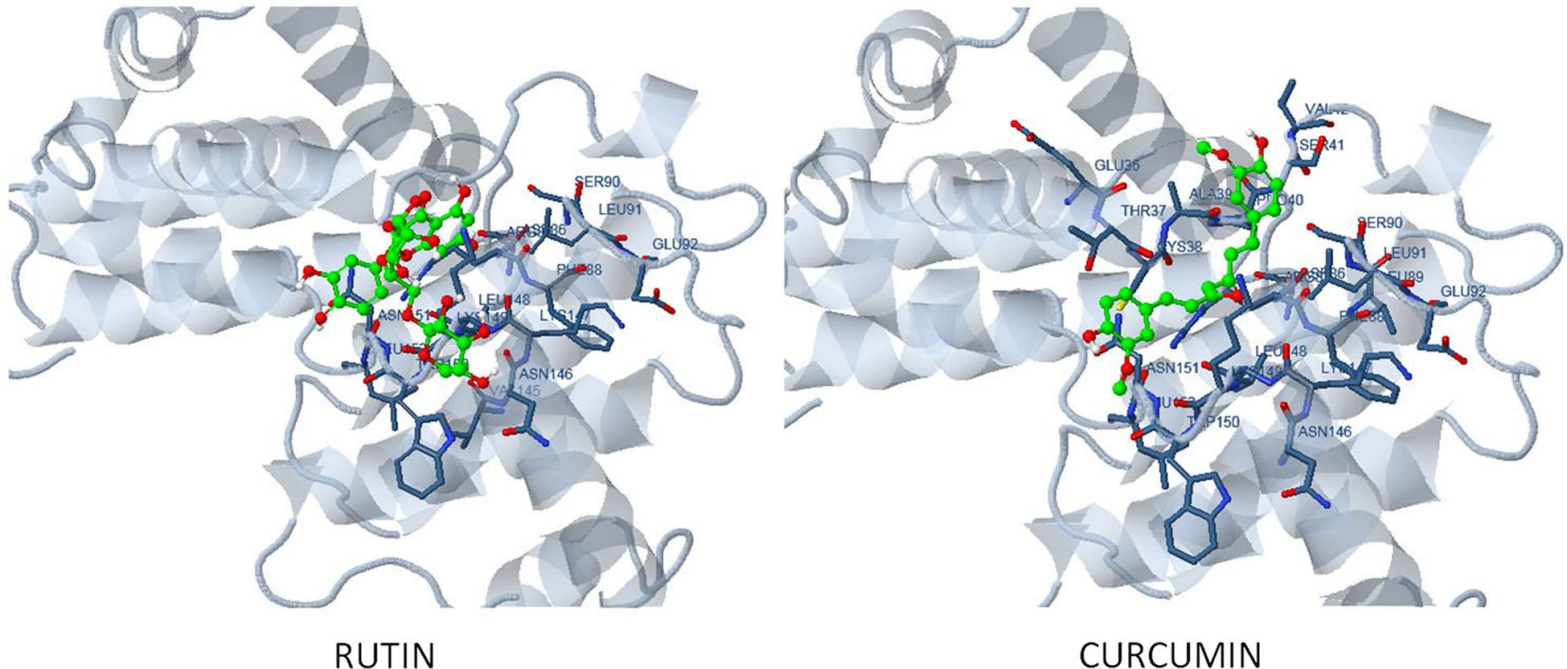
Molecular docking conformation and interactions of rutin and curcumin with Cyclin D1

**Fig. 8:**
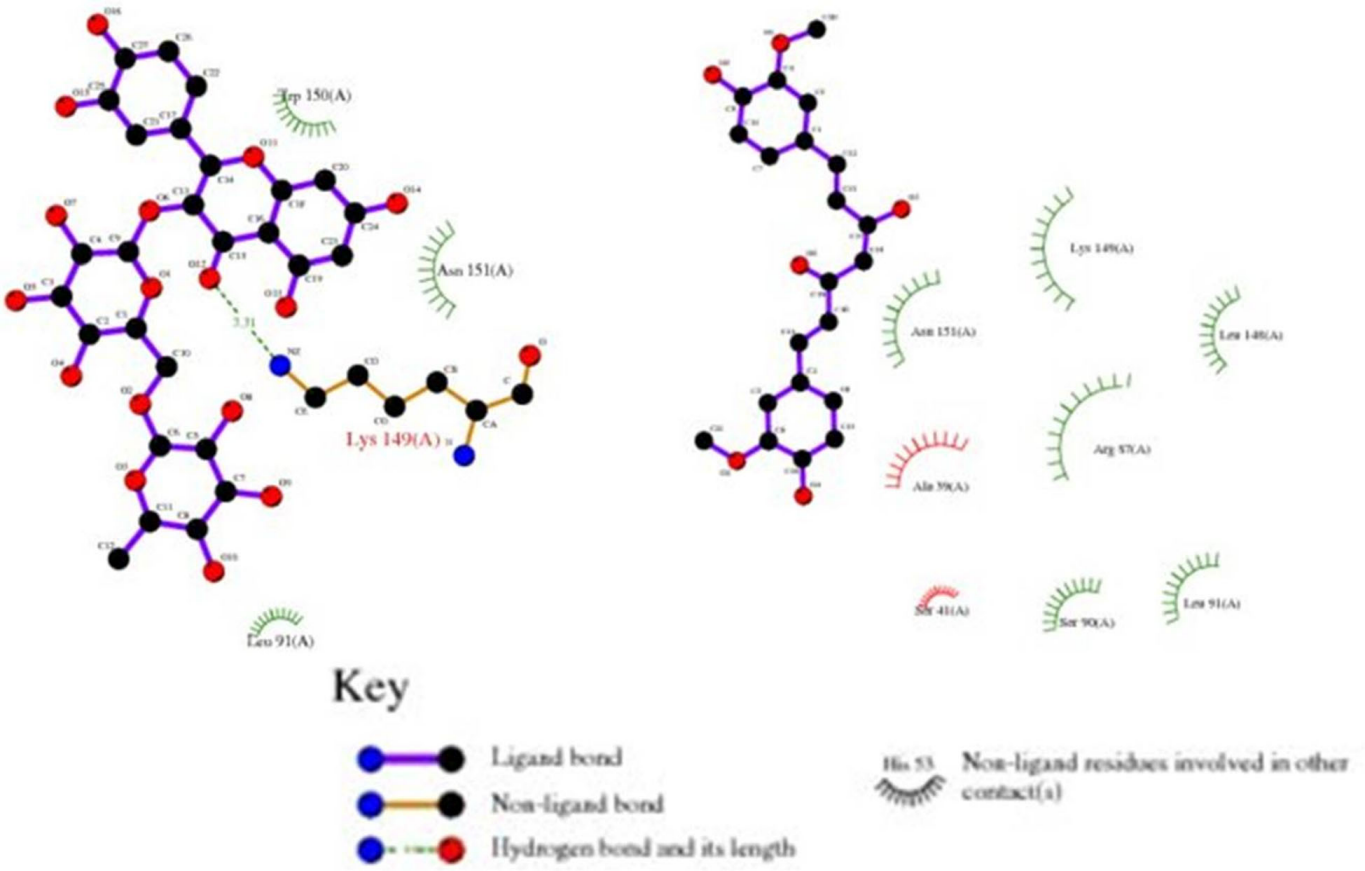
Two-dimensional plot showing the primary interacting residues of Cyclin D1

Molecular docking studies predicted good interactions between three dimensional structure of drug target (CCND1, PDBID: 2w96) and selected ligands; rutin and curcumin. Molecular docking revealed that both the compounds are able to bind in the ligand binding domain. *In silico* docking studies revealed interaction of two active compounds with the common vital ligand binding site residues (Leu91, Lys149, Asn151) of cyclin D1. Both rutin and curcumin docked at a common ligand binding site of CCND1 slightly varied intensity as estimated by their size, structure, stereochemistry (Figs. 7 and 8; Table 4). We also examined their complete interaction profile including hydrogen bonds, HB plot, polar, hydrophobic, pi-pi and cation-pi interactions. The estimated free energy of binding with Cyclin D1 for rutin was −4.26 kcal/mol and for curcumin was −4.67 kcal/mol which is very similar, however, the estimated inhibition constant (Ki) was 757.57 μM and 380.02 μM respectively.

**Table 4:** Docking features and values for rutin and curcumin

Features	RUTIN	CURCUMIN
Est. Free energy of binding	−4.26 kcal/mol	−4.67 kcal/mol
Est. Inhibition Constant, Ki	757.57 μM	380.02 μM
vdW + Hbond + desolv Energy	−5.43 kcal/mol	−6.37 kcal/mol
Electrostatic energy	−0.01 kcal/mol	−0.12 kcal/mol
Total intermolecular energy	−5.44 kcal/mol	−6.49 kcal/mol
Interaction surface	653.668	684.416

## Discussion

RCC is a complex heterogeneous tumors involving altered genes and proteins. We performed a transcriptional profiling and functional analysis of RCC to understand the role of identified significant genes in regulation of physiological processes through biological pathways/networks. We found CCND1 as one of the significantly expressed gene and potential biomarker RCC.

HEY1, an upregulated gene, has been reported to be mediator of notch signaling, showing pro-oncogenic function and promotes cancer progression [45, 46]. Neuropilin-2 (Nrp2) is a well known receptor for the vascular endothelial growth factor-C (VEGF-C) and activates lymph nodes as well as promotes tumor metastasis by lymphangiogenesis [47, 48]. LEF1 interacts with β-catenin and plays critical role in proliferation of RCC by activating downstream target genes [49, 50]. Wnt/β-catenin signaling, found activated, regulates embryonic development and is involved in many diseases including cancer, polycystic kidney disease [51–54]. WNT signal and its paracrine mode to growth of cancer cells makes it clinically important to understand the metastasis of tumor cells [53, 55, 56]. HIST1H3H is frequently altered chromatin factors in many cancers [57, 58]. Aldolase, a family member of glycolysis enzymes, was found to be significantly affecting RCC. Aldolase-A was reportedly upregulated while aldolase-B was downregulated in RCC and human primary hepatocellular carcinoma [59–62]. SLC12 family members are involved in regulation of cell volume, blood pressure and chloride concentration, and play a critical role in diseases like cancer, epilepsy and osteoporosis [63]. In the present study, SLC12 was down regulated that is in accordance to other findings [64]. CALB1 is reported to be altered in RCC and found to be negatively stained compared to normal tissue [61, 65].

CCND1 was overexpressed in our as well as other transcriptomics studies [66–69]. We validated CCND1 overexpression by using tissue microarray platform and *in silico* docking analysis was done to check its therapeutic potential as it plays a key role in G1-S phase transition of cell cycle. There are reports of anti-proliferative, apoptosis inducing and chemopreventive effects of natural bioactive flavonoids like baicalein, catechin, genistein, quercetin, and rutin. Docking analysis showed that rutin and curcumin binds to CCND1 and can potentially inhibit downstream CCND1/CDK4/CDK6 complex formation, required for G1-S phase transition. Our finding demonstrate the anticancer drug targets potential of CCND1 and rutin and curcumin as potential inhibitors, however, this *in silico* docking study has to be validated further.

## Conclusion

Our microarray and immunohistochemistry results suggest significantly high levels of cyclin D1 expression in RCC. Distinct transcriptomic signatures identified for RCC needs verification at larger dataset and additional significant genes need to be further validated for identification of novel biomarkers. The critical role of CCND1 in RCC metastasis by activating G1-S transition of cell cycle has drawn our attention to examine its potential as anticancer drug target. Our *in silico* docking study shown CCND1 protein as an attractive anticancer target and natural flavanoids rutin and curcumin as potential anticancer drug of RCC and they may be promising in the prevention of kidney cancer too. Quantitative structure-activity relationship studies, ligand binding, efficacy and toxicity should be further investigated before clinical trials. Clinical and therapeutic applications of these natural ligands were initially limited by their low solubility and bioavailability but combination with adjuvant and nano-technology based delivery vehicles can immensely improve their potential. Moreover, these are reported to act in synergism with several other natural compounds or synthetic agents routinely used in chemotherapy and can assist in cancer prevention and treatment when used alone or in combination with other conventional treatments.

## Additional file

Additional file 1: Differentially expressed genes of RCC from Saudi patients on comparing RCC with normal kidney tissue. Transcriptomics profiling revealed 1490 differentially expressed genes, 1034 up-regulated and 456 down-regulated, with fold change ≥ 2 and *p*-value < 0.05. (XLSX 209 kb)
